# Edge Computing for Effective and Efficient Traffic Characterization

**DOI:** 10.3390/s23239385

**Published:** 2023-11-24

**Authors:** Asif Khan, Khurram S. Khattak, Zawar H. Khan, Thomas Aaron Gulliver, Abdullah

**Affiliations:** 1 Department of Electrical and Computer Engineering, University of Victoria, Victoria, BC V8W 2Y2, Canada; asifk@uvic.ca (A.K.); khanz@uvic.ca (Z.H.K.); agullive@ece.uvic.ca (T.A.G.); 2 Department of Computer Systems Engineering, University of Engineering and Technology (UET), Peshawar 25000, Pakistan; 3 National Center for Big Data and Cloud Computing, University of Engineering and Technology (UET), Peshawar 25000, Pakistan

**Keywords:** edge computing, traffic monitoring, urban mobility, Internet of Things, Raspberry Pi, vehicle detection

## Abstract

Traffic flow analysis is essential to develop smart urban mobility solutions. Although numerous tools have been proposed, they employ only a small number of parameters. To overcome this limitation, an edge computing solution is proposed based on nine traffic parameters, namely, vehicle count, direction, speed, and type, flow, peak hour factor, density, time headway, and distance headway. The proposed low-cost solution is easy to deploy and maintain. The sensor node is comprised of a Raspberry Pi 4, Pi camera, Intel Movidius Neural Compute Stick 2, Xiaomi MI Power Bank, and Zong 4G Bolt+. Pre-trained models from the OpenVINO Toolkit are employed for vehicle detection and classification, and a centroid tracking algorithm is used to estimate vehicle speed. The measured traffic parameters are transmitted to the ThingSpeak cloud platform via 4G. The proposed solution was field-tested for one week (7 h/day), with approximately 10,000 vehicles per day. The count, classification, and speed accuracies obtained were 79.8%, 93.2%, and 82.9%, respectively. The sensor node can operate for approximately 8 h with a 10,000 mAh power bank and the required data bandwidth is 1.5 MB/h. The proposed edge computing solution overcomes the limitations of existing traffic monitoring systems and can work in hostile environments.

## 1. Introduction

Urbanization is expected to drive global economic growth in the coming decades by increasing productivity and reducing poverty. However, this growth is threatened by challenges to urban mobility including greenhouse gas (GHG) emissions and lost productivity due to road accidents and congestion. The road transportation sector accounts for 25% of worldwide fuel consumption and 29% of GHG emissions [1]. Traffic congestion also results in a significant reduction in productivity, with an average driver in the USA losing 36 h and USD 564 in 2021 [2]. Moreover, according to the World Health Organization (WHO), road accidents cause 1.3 million deaths and 50 million non-fatal injuries every year [3]. Therefore, it is imperative to develop innovative and effective solutions such as intelligent transportation systems (ITS) to mitigate these challenges and ensure efficient urban mobility.

ITS-based solutions are a promising means of improving road network efficiency. Detailed traffic data including vehicle count, speed, and classification, flow, spatial/temporal densities, vertical/horizontal headways, road capacity, heatmaps, and trajectories are essential to provide insights for traffic engineers to improve transport network management. Furthermore, these parameters can be employed in traffic simulation software [4,5] to aid urban planners in designing effective road networks.

Both intrusive and non-intrusive traffic monitoring systems have been developed. However, these solutions have limitations, including only measuring traffic count and speed, installation and maintenance difficulties, and high costs [6]. With advancements in image processing techniques, roadside video can now be employed. While Internet-of-Video-Things (IoVT) solutions are effective, the high bandwidth requirements for roadside video transmission to servers are a major limitation [6,7]. Image processing edge computing solutions have been proposed to overcome this problem. However, the computational power of devices such as Raspberry Pi (RPi) limits the ability to provide detailed traffic information. Existing edge computing solutions provide either count [1,8,9,10], count and speed [11,12,13,14], or count and classification [11,15,16,17,18].

In this work, an edge computing solution is proposed to overcome the limitations of existing traffic monitoring systems. The objective is to accurately obtain vehicle count, speed, type, and direction, flow, peak hour factor, density, time headway, and distance headway. This is achieved using a sensor node composed of a RPi 4, Pi camera, Intel Movidius Neural Compute Stick 2, Xiaomi MI 10,000 mAh power bank, and Zong 4G Bolt+. The pre-trained Mobilenet-SSD model from the Intel OpenVINO Toolkit is employed for vehicle count and classification [19]. Vehicle speed is estimated using a centroid tracking algorithm running on the RPi 4. The measured traffic parameters are transmitted to the ThingSpeak cloud platform using 4G. Then, the data archived in ThingSpeak can be used for traffic flow analysis to aid in transportation network planning and management.

The remainder of this paper is organized as follows. Section 2 provides an overview of related work in the area. Section 3 presents the architecture of the proposed system, including the hardware components and software algorithms used. Section 4 gives some experimental results including the accuracy and reliability of the proposed system. Finally, Section 5 provides some concluding remarks and suggestions for future research.

## 2. Related Work

The development of intelligent mobility solutions requires accurate real-world traffic data. While computer vision-based approaches have been shown to be better than intrusive and non-intrusive sensor-based solutions [6], their capabilities are limited, primarily due to a lack of computational resources. Existing edge computing solutions provide either vehicle count, count and classification, or count and speed.

### 2.1. Count

An edge computing solution based on an RPi and web camera was presented in [9] which achieves a vehicle count accuracy of 83%. Vehicle count, road density, time headway, and vehicle emissions were obtained with the system in [1]. This solution used an RPi 4 and Pi camera with four sensors to measure carbon monoxide, carbon dioxide, and particulate matter. A vehicle count accuracy of 86% was reported and the measured parameters were transmitted to the ThingSpeak cloud platform using the RPi Wi-Fi module.

### 2.2. Count and Classification

In [15], a system to count and classify vehicles at a highway toll booth was developed using an RPi B and Pi camera [8]. In [10], an edge computing solution to count and classify vehicles was presented which employs an RPi 2, Pi camera, and MySQL web server database [17]. In [16], an RPi B and Samsung smart security camera were used to transmit parameters to a remote web server for display. A vehicle count accuracy of 83% was obtained. In [18], an edge computing solution was developed using an RPi 2 and Pi camera to count vehicles and classify them as small or large. The data were stored locally on the RPi 2 for archiving purposes. In [20], a real-time stereo vision system was presented to count vehicles and classify them as cars or small or big trucks. It employs an RPi 3B and USB webcam and transmits the parameters to a local web server for display.

### 2.3. Count and Speed

A solution using an RPi 2B+ and Pi camera to count vehicles and estimate their speed was given in [12]. The Flask web framework was used to archive the parameters on an edge cloud server. In [21], an edge computing solution to count vehicles and estimate their speed was presented which uses an RPi 2 and Pi camera. The effect of different frame sizes on the CPU and memory was examined. It was found that the CPU performance was not significantly affected by the frame size, but higher-resolution frames required more memory. In [22], a system to count vehicles and estimate their speed was developed which uses an RPi 3B and Pi camera. The parameters were stored locally on the RPi, and a count accuracy of 100% and speed accuracy of 90% were reported. Another solution was proposed in [13] which uses an RPi 3B and Pi camera. All of these edge computing solutions are limited by the RPi computing resources. Thus, an edge computing solution is proposed here to overcome this constraint. The advantages of the proposed solution are as follows.

- Existing edge computing solutions only measure two traffic parameters, either vehicle count and classification [15,16,17,18,20,23] or vehicle count and speed [5,12,13,21,22]. Conversely, the proposed solution can measure nine traffic parameters, namely, vehicle count, speed, direction, and type, flow, peak hour factor, density, time headway, and distance headway.
- The proposed solution can classify five different types of vehicles, namely, cars, buses, motorbikes, bicycles, and animal-drawn carts (horse and cow). This is greater than the number of vehicle classes provided by existing solutions [15,16,17,18,20].
- The proposed solution can count and classify vehicles with an accuracy of 93%, which is better than the accuracy reported in previous studies [1,4,9,14,16,22].
- The proposed solution can count and estimate the speed of a wide variety of vehicles as pedestrians. This includes trams (trains), airplanes, and boats. This is because the detection model was trained on over 70 different objects, including these vehicles. Vehicle direction is also obtained. This makes the proposed system ideal for characterizing heterogeneous traffic behavior. Note that no other system provides the direction of vehicles.
- The proposed solution was designed considering cost, reliability, and scalability. The sensor node costs less than USD 300 and has a low power consumption of 1.2 A per hour. Unlike previous systems, the proposed solution transmits the measured parameters to a cloud platform using 4G with a data bandwidth requirement of approximately 1.5 MB per hour.

## 3. System Architecture

**Figure 1:**
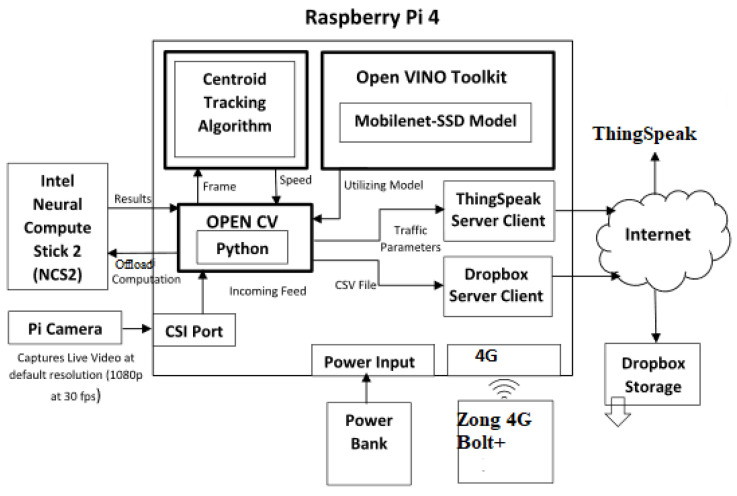
The architecture of the proposed system.

The proposed edge computing solution for real-time traffic characterization is shown in Figure 1. It measures nine traffic parameters and transmits them to a cloud platform using the Zong 4G Bolt+. The system comprises three main components: (1) sensor node, (2) computer vision module, and (3) cloud platform.

### 3.1. Sensor Node

The sensor node was fabricated using cost-effective but powerful hardware components. This includes an RPi 4 (a low-cost Linux-based single-board computer) and a Pi camera v2 connected via the Camera Serial Interface (CSI) port. It can capture roadside video at 20 frames per second (fps) with 1080 p resolution. A 10,000 mAh Xiaomi Mi Power Bank was used to provide sufficient battery life for extended use. The sensor node was designed for edge deployment and only consumes 1.2 A per hour measured using a Keweisi USB power tester. The measured traffic parameters were transmitted to the cloud platform using a Zong 4G Bolt+. This provided reliable and efficient communications with low complexity.

To overcome the computing limitations of a single-board computer such as RPi [24], the proposed solution employed an Intel Movidius Compute Stick 2. This was designed to provide computation power to edge devices. It has a Myriad X visual processing unit and a dedicated hardware accelerator for artificial intelligence and computer vision applications [25]. This enables the offloading of complex computations from the RPi and consumes much less power.

### 3.2. Computation Workflow

**Figure 2:**
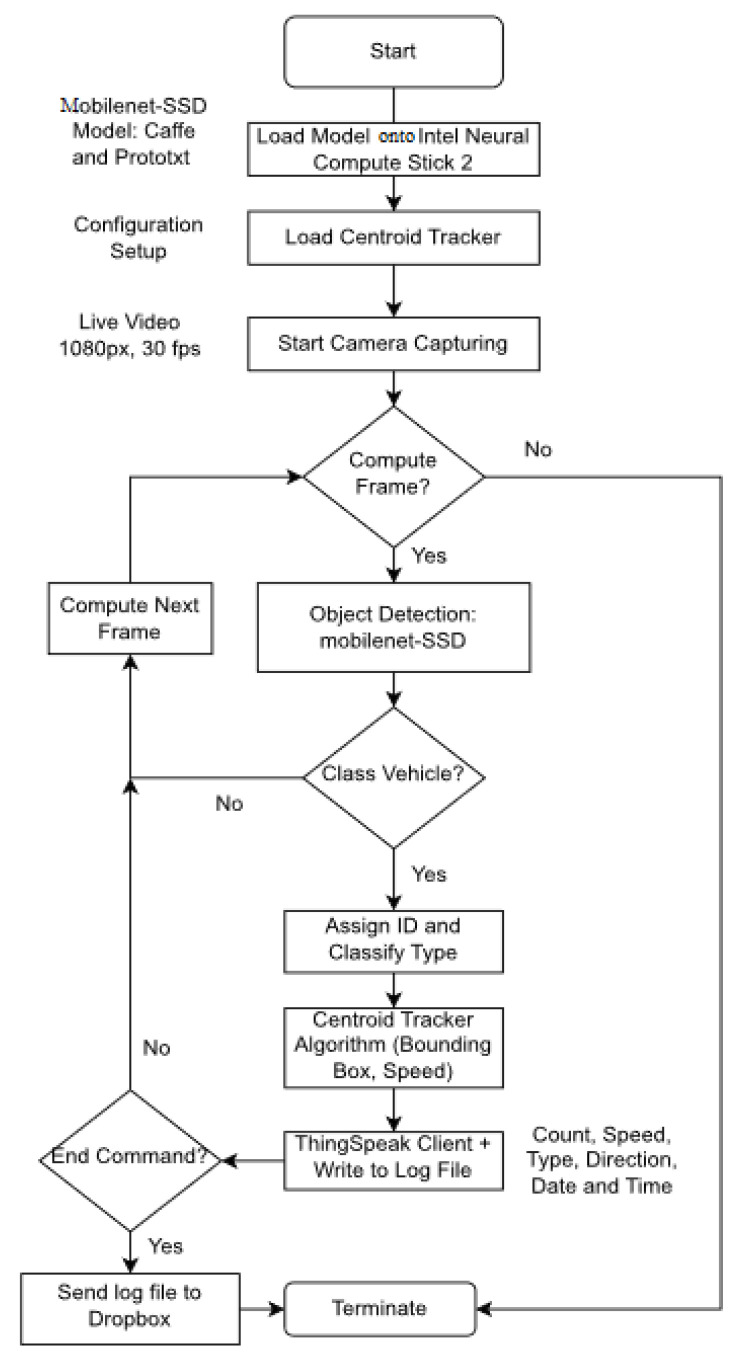
The system computation workflow.

The computation workflow for the sensor node is shown in Figure 2. It includes video capture, preprocessing (including blob extraction, scaling, and resizing), and traffic parameter extraction tasks implemented in Python using the free open-source image processing library OpenCV. The workflow steps are as follows.

1. A Python script is executed to load the pre-trained Mobilenet Single Shot Detector (Mobilenet-SSD) model onto the compute stick. This is part of the OpenVINO Toolkit which is a deep learning model for detecting objects from an image or video. Compared to YOLO and Faster R-CNN, Mobilenet-SSD is better suited for resource-constrained devices and provides good real-time object detection accuracy [26]. The Mobilenet-SSD model consists of two components, namely, the backbone model and the SSD head. The backbone model classifies objects using an image classification network for feature extraction, while the SSD head is a convolution layer that creates bounding boxes around the detected objects [27]. The proposed algorithm uses SSD and Mobilenet to detect vehicles using a pre-trained Caffe model [28]. The prototxt prototype machine learning (ML) model is loaded onto the Neural Compute Stick for use with the Caffe framework [7]. This model has been trained on over 70 objects, but the focus here is on the classification of vehicles as cars, buses, motorbikes, bicycles, or animal-drawn carts.
2. The parameters for the centroid tracking (CR) algorithm are first loaded from a configuration file to the RPi. This is an algorithm in OpenCV used for object tracking in video using the Euclidean distance between pixels in consecutive frames [29]. The CR algorithm can be used in combination with object detection models to calculate and store object coordinates. In this work, the Mobilenet-SSD model is used to obtain vehicle coordinates. The distance traveled by a vehicle is estimated using the difference between coordinates in successive video frames, and this is used to estimate the speed as distance divided by time.
3. The roadside video is captured at 30 fps and 1080p resolution using the OpenCV video capture function. The detection model and CR algorithm are used to lower the frame resolution. Although a higher resolution and fps may improve accuracy [30], due to computational constraints, the maximum possible resolution is 360p at 20 fps.
4. The video frames are processed individually. First, a frame is checked to determine if it has already been processed. If not, it is passed through the Mobilenet-SSD model for object detection and classification.
5. If a vehicle is detected in the video frame, it is classified as either a car, bus, motorcycle, bike, or animal-drawn cart, and is assigned a unique ID. The frame is then passed to the CR algorithm to estimate speed.
6. After object classification and speed estimation, the results are sent in real-time to ThingSpeak and simultaneously written to a log file. This process is repeated until a termination command is issued. Upon termination, the log file is transmitted to Dropbox, and execution is stopped.

### 3.3. Cloud Platform

**Figure 3:**
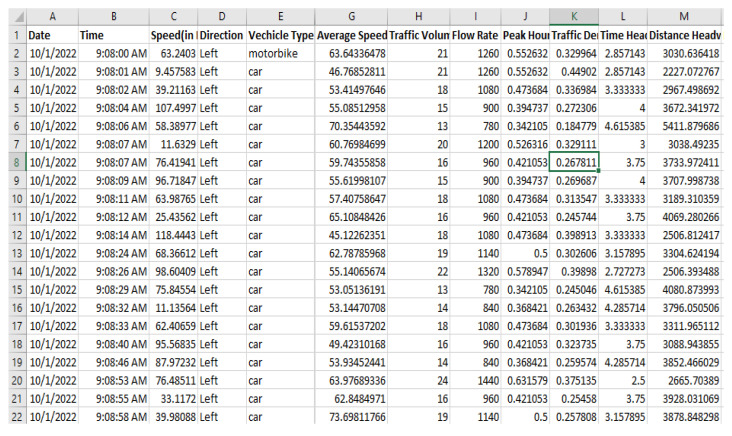
Real-time traffic parameters from the sensor node for the road under observation.

ThingSpeak is a cloud platform that facilitates communication with Internet-enabled edge devices through APIs. The platform is free and open-source. The ThingSpeak libraries are installed on the sensor node RPi. The ThingSpeak client transmits vehicle count, speed, direction, and type, flow, peak hour factor, density, time headway, and distance headway every 15 s. An example of the data for the road under observation is shown in Figure 3.

To ensure data reliability, traffic parameters are stored in a log file in the RPi system root directory called log.csv. Every time a vehicle is detected, a record is added to this file that includes the date (year, month, and day), time, vehicle type, speed, and direction. This serves as a backup of the traffic data that can be referred to in the future if necessary. In addition, at the end of the observation period, the log file is uploaded to Dropbox which is a cloud file-sharing and archiving platform. This allows for remote sharing and access of traffic data from Dropbox servers, providing greater flexibility and convenience.

In designing the proposed solution, a key consideration was low operational costs. To achieve this, efforts were made to minimize data bandwidth requirements. Edge computing solutions have a distinct advantage over IoVT-based solutions as they reduce the transmission bandwidth, resulting in lower costs [13].

To examine data bandwidth requirements for the proposed solution, the sensor node was connected through a Huawei Nova 3i smartphone Wi-Fi hotspot. The bandwidth used by the node was estimated to be 1.5 MB per hour. In Pakistan, a standard monthly 5 GB 4G internet data package costs approximately USD 15, so the proposed solution can transmit traffic data to the cloud platform for approximately 3300 h with this package.

## 4. Performance Results

**Figure 4:**
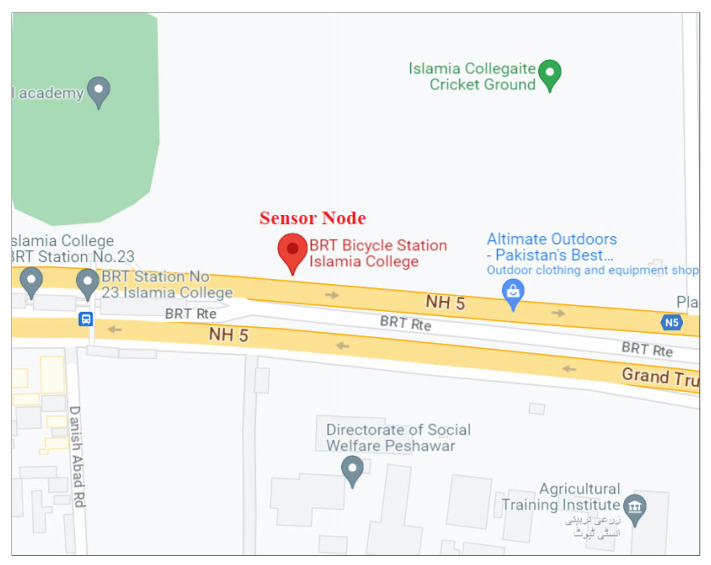
Google Maps location of the sensor node.

**Figure 5:**
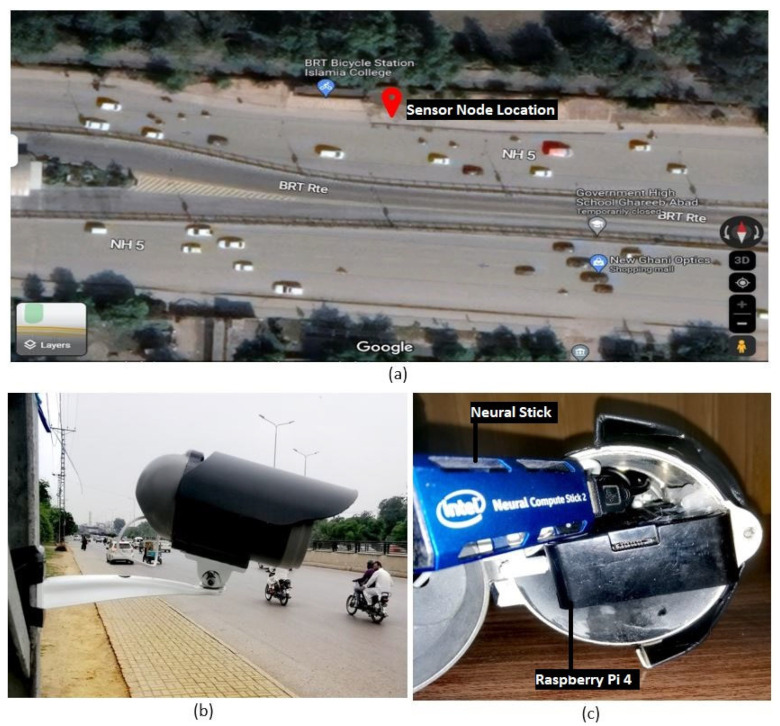
Sensor node installation: (**a**) overhead location, (**b**) field view, and (**c**) inner view.

University Road in Peshawar, Pakistan was chosen to evaluate the system. This is the main arterial road in the city. The sensor node was installed south of Islamia College on the north side of the Bus Rapid Transport (BRT) station as shown in Figure 4. The GPS coordinates of the installation location are 33.99841270487691 latitude and 71.47962908395094 longitude. This road connects major institutions such as universities and government organizations, making it the most heavily traversed route in Peshawar. It is a bidirectional road, but the sensor node was installed on the side where traffic runs west to east to prevent direct sunlight on the camera which would compromise the data obtained. The sensor node was positioned perpendicular to the traffic flow as illustrated in Figure 5. It was used to collect data for seven days from Monday, 10 January 2022 to Sunday, 16 January 2022. Each day, the node was in operation for seven hours from 9:00 AM to 4:00 PM.

Throughout the evaluation period, the sensor node performed reliably with no issues such as power interruptions or system crashes. It successfully captured traffic data as shown in Table 1. Each day, about 10,000 vehicles traversed the road segment during the seven hours from 9:00 AM to 4:00 PM, and over the week, 69,285 vehicles were observed. The majority of the vehicles were cars (81.0%), followed by motorcycles (15.6%), buses (2.5%), bicycles (0.72%), and animal-drawn carts (0.16%). The highest and lowest traffic volumes were on Monday and Thursday, respectively, as shown in Table 1.

### 4.1. Sensor Node Accuracy

Accurate estimation of traffic parameters is critical for an effective monitoring system. In this section, the vehicle count and speed obtained were examined to evaluate the proposed solution. The accuracy was determined for a randomly selected one-hour period from 9:00 AM to 10:00 AM on Saturday, 15 January 2022. The corresponding video was manually analyzed to determine the vehicle count, type, and speed, and these results were compared with those obtained using the sensor node. There were 2356 vehicles in the manual count but only 2196 vehicles with the node. Thus, the system count accuracy was 93.2%, as indicated in Table 2. The mean average precision (mAP) for object classification was 79.8%, as determined from the model results and verified by the manual count from the one hour of video. This is higher than with other object detection models designed for edge devices [31]. The speed of each vehicle was manually determined by measuring the time it took to travel a given distance. These results were compared with those from the sensor node. Table 2 shows that the speed estimation accuracy was 82.9%. The only other speed accuracy result reported in the literature was 90% [22], but this was for a single vehicle measured between two locations.

#### 4.1.1. Regression Modeling

**Figure 6:**
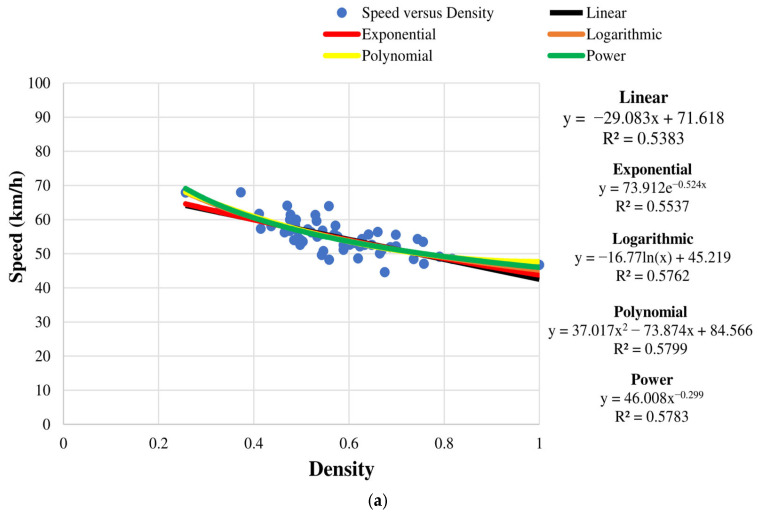

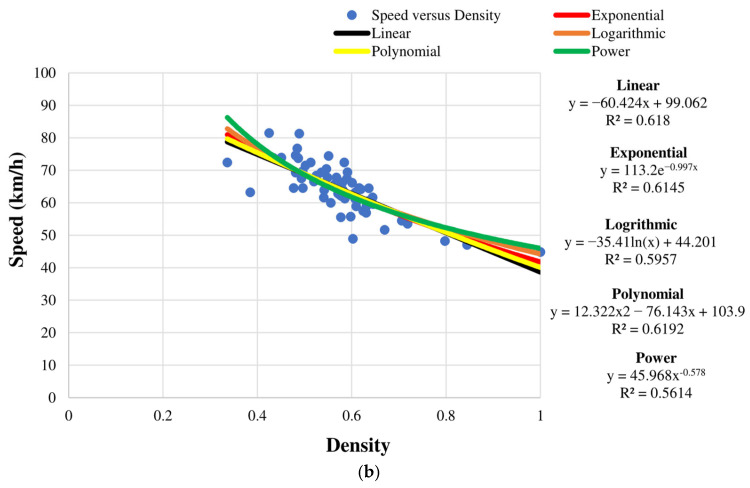
Speed versus density over one hour obtained (**a**) manually and (**b**) from the sensor node.

The Greenshields traffic flow model [32] was used to predict the relationship between traffic speed and density on a road. According to this model, an increase in density results in an increase in speed until the road reaches capacity (100% density). At capacity, the speed drops to 0 and the road is considered to be in a jammed state. To verify the accuracy of the sensor node, the relationship between speed and density was modeled using one hour of data, and the results are shown in Figure 6. The models employed are exponential, linear, logarithmic, polynomial, and power. The corresponding R^2^ values indicate how well the data fit the model and range from 0 to 1, with smaller values indicating a better fit. These results show that a second-order polynomial is the best fit. The corresponding polynomials for the sensor node and manual observations are:

(1) $$y1=\;12.32x^{2}\;-76.14x+103.9$$

and

(2) $$y2=\;37.02x^{2}\;-\;73.87x+84.57,$$

respectively. The error between (1) and (2) is given by:

(3) $$\frac{\left|\left|y2\right|\right|-\;\left|\left|y1\right|\right|}{\left|\left|y2\right|\right|}$$

where

(4) $$\left|\left|y1\right|\right|=\sqrt{{y1}_{1}{+y1}_{2}{+y1}_{3}{+\ldots +y1}_{n}}\;\;\;\;\;$$

(5) $$\left|\left|y2\right|\right|=\sqrt{{y1}_{1}{+y1}_{2}{+y1}_{3}{+\ldots +y1}_{n}}\;.\;\;\;\;\;\;$$

This gives an error of 17.1%.

#### 4.1.2. Limitations and Challenges

The system developed has undergone rigorous testing to evaluate its accuracy and efficiency. This revealed some limitations and challenges which are discussed below.

1. Camera orientation is a significant factor affecting sensor node accuracy. Thus, the camera should be oriented to best capture vehicles passing through the area of interest. It was observed that smaller vehicles are sometimes masked by larger vehicles, resulting in missed detections.
2. Although other ML models may provide more accurate vehicle classification, the proposed solution employs Mobilenet-SS due to its computational efficiency [33]. The model is specifically designed for edge computing solutions, and its lightweight computational footprint makes it a good choice for the system.
3. The choice of video resolution and fps can significantly affect the accuracy. However, a higher video resolution and fps result in greater computational requirements. Considering this tradeoff, 20 fps is used which resulted in larger errors compared to 25 fps [31], e.g., greater Euclidean distance errors with the CR algorithm [34].
4. The proposed sensor node was tested on a busy road with heterogeneous traffic where vehicles do not follow lane discipline. This behavior will increase the error in calculating speed and other parameters. Therefore, the accuracy of the sensor node results will vary depending on the traffic environment.

Note that despite these limitations and challenges, the proposed solution provides a reliable and efficient means of counting vehicles and estimating their speed.

### 4.2. Traffic Parameters

The proposed solution utilizes computer vision algorithms to obtain vehicle count, type, direction, and speed. The remaining traffic parameters, namely, flow, peak hour factor, density, time headway, and distance headway are obtained as follows. Flow is the number of vehicles that pass a point on a road per unit of time and is given by:

(6) $$q=\frac{n}{t}$$

where *n* is the number of vehicles in time *t*. The peak hour factor is a measure of the traffic flow during a particular hour $q_{60}$ relative to the highest flow in a 15-min period $q_{15}$ on a road segment and is given by:

(7) $$PHF=\frac{q_{60}}{q_{15}}$$

Traffic density is the number of vehicles per unit length of the road and is given by:

(8) $$k=\frac{q}{v}$$

Time headway is the distance between consecutive vehicles and can be expressed as:

(9) $$h=\frac{1}{q}$$

Distance headway is the distance between consecutive vehicles and is given by:

(10) $$s=\frac{1}{k}$$

The speed, flow, and density for each of the seven days are given in Table 3. These results are for one-minute intervals. The minimum speed ranges between 6.1 km/h and 35.7 km/h, the maximum speed between 74.1 km/h and 89.7 km/h, and the mean speed between 50.3 km/h and 61.3 km/h. The minimum flow is between 3.0 veh/m and 12.0 veh/m, the maximum flow is between 32.0 veh/m and 39.0 veh/m, and the mean flow is between 16.6 veh/m and 24.1 veh/m. The standard deviation of the flow is between 3.8 veh/m and 4.9 veh/m, indicating the differences in flow each day. The density ranges from a minimum of 3.9 veh/km to a maximum of 56.9 veh/km. The mean density is between 20.2 veh/km and 27.6 veh/km, and the standard deviation is between 5.5 veh/km and 7.4 veh/km, so the variations each day are similar.

**Figure 7:**
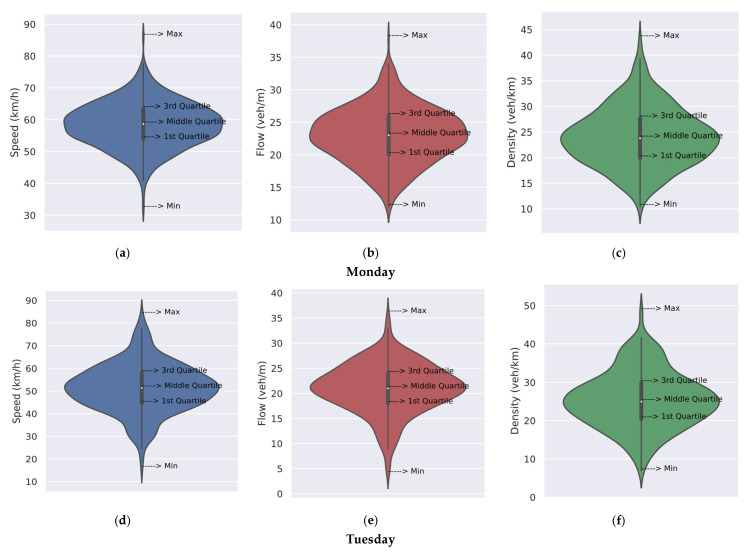

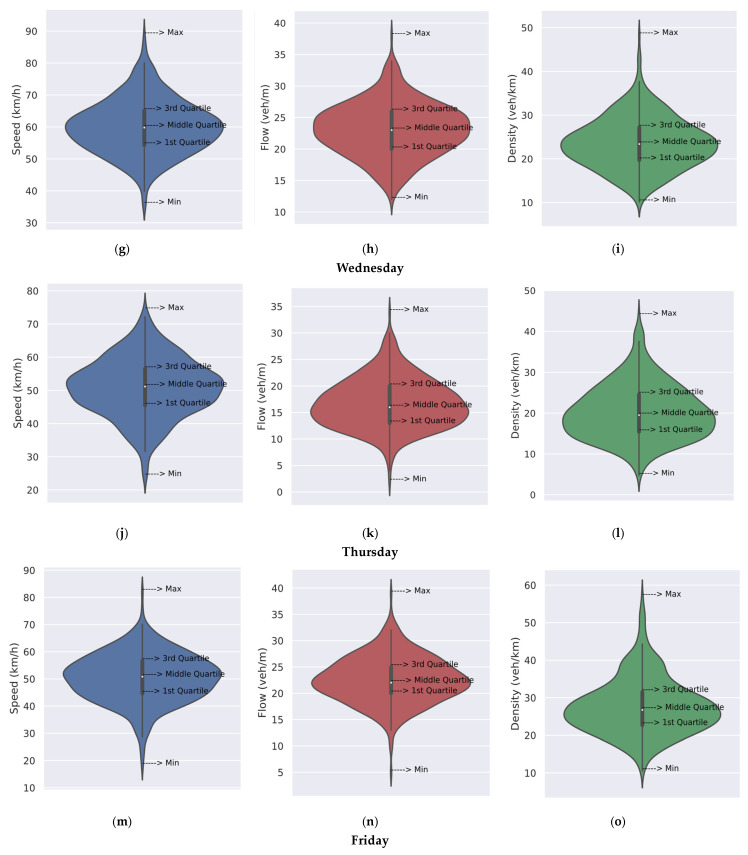

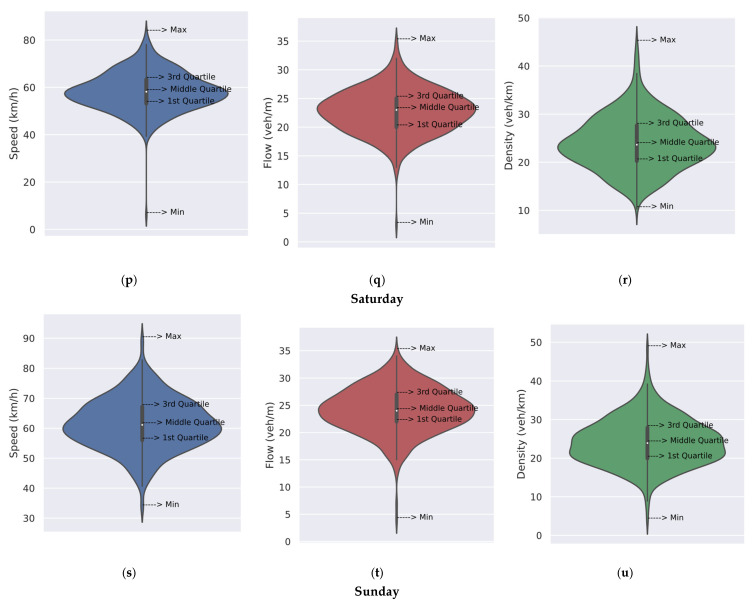
Interquartile representation of speed (blue), flow (red), and density (green) using violin plots for the week 10–16 January 2022: (**a**–**c**) Monday, 10 January 2022; (**d**–**f**) Tuesday, 11 January 2022; (**g**–**i**) Wednesday, 12 January 2022; (**j**–**l**) Thursday, 13 January 2022; (**m**–**o**) Friday, 14 January 2022; (**p**–**r**) Saturday, 15 January 2022; and (**s**–**u**) Sunday, 16 January 2022.

Figure 7 presents violin plots for the speed, flow, and density results. They combine the benefits of both box plots and kernel density plots and allow for easy interpretation of the data distributions by quartile regions. Figure 7a gives the speed for Monday, 10 January 2022. The plot is wide in the middle which indicates that the speed of most vehicles is concentrated around 58.4 km/h (mean), between 53.9 km/h (first quartile) and 63.4 km/h (third quartile). The plot is narrow at the extreme points, 32.1 km/h (minimum) and 86.1 km/h (maximum), so there are very few vehicles at these speeds. Figure 7b,c gives the corresponding flow and density plots. They indicate that the flow and density of most vehicles are between 20.0 veh/m and 24.0 veh/m and 19.9 veh/km and 27.7 veh/km, respectively. Similar results were obtained for Tuesday, 11 January 2022 to Friday, 14 January 2022, as shown in Figure 7d–u. For example, the speed is between 44.6 km/h and 67.1 km/h for most vehicles as the plots are wide in the middle. Figure 7p–u gives the plots for the weekend (Saturday and Sunday). These plots are smaller than on the weekdays which indicates lower traffic volumes. This is reflected in the higher speeds, mostly between 53.3 km/h and 67.1 km/h. The means are also higher, so traffic conditions are better on these days. Further, the maximum speed of 89.7 km/h occurred on Sunday.

### 4.3. Traffic Behavior Analysis

The Greenshields model is widely used to characterize uninterrupted traffic flow conditions [32]. It defines the relationship between speed, density, and flow. The speed is given by:

(11) $$v\left(k\right)=vf\left(\;1-\frac{k}{k_{j}}\;\right)\;$$

where *vf* is the free flow speed when the density is zero, *k* is the density, and *k_j_* is the jam density (when the speed is zero). The flow can be expressed as:

(12) q=kv

Substituting (11) in (12) gives the relationship between flow and density as:

(13) $$q\left(k\right)=vf\;\left(k-\frac{k^{2}}{k_{j}}\;\right)$$

The relationship between flow and speed is then:

(14) $$q\left(v\right){=k}_{j}v-\frac{v^{2}}{vf}$$

In this paper, parameters for vehicles on the road segment were obtained for one-minute intervals, e.g., veh/min. These data were used to model the relationships between density and speed, density and flow, and speed and flow using exponential, linear, logarithmic, polynomial, and power expressions. The corresponding *R*^2^ values indicate that the linear model is best as there is no significant difference between the values.

#### 4.3.1. Density versus Speed

**Figure 8:**
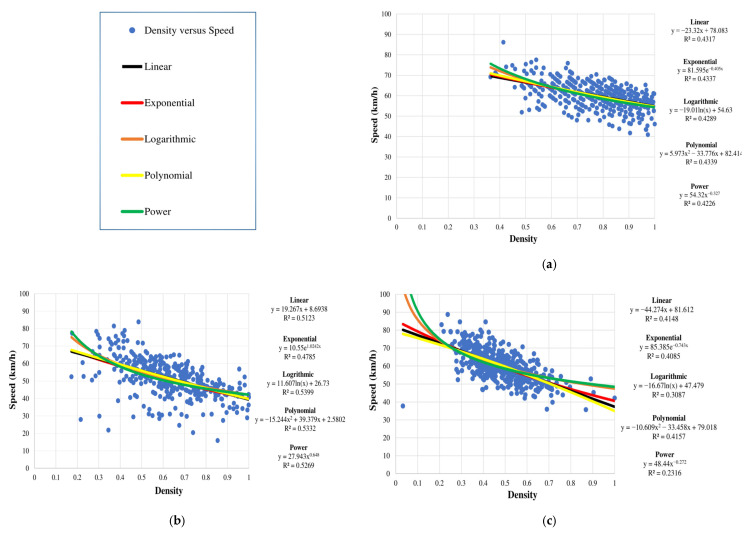

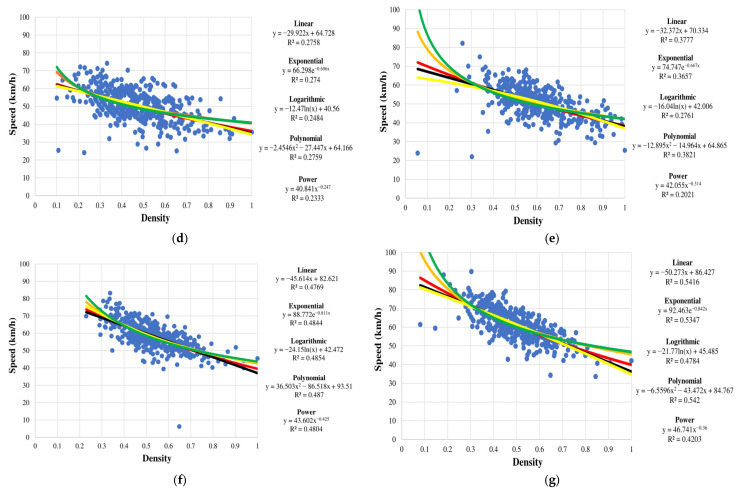
Speed versus density for the week 10 January 2022 to 15 January 2022: (**a**) Monday, 10 January 2022; (**b**) Tuesday, 11 January 2022; (**c**) Wednesday, 12 January 2022; (**d**) Thursday, 13 January 2022; (**e**) Friday, 14 January 2022; (**f**) Saturday, 15 January 2022; and (**g**) Sunday, 16 January 2022.

The relationships between density and speed for the seven days are given in Figure 8. These results suggest that the flow on the road is good throughout the week, with the average speed never low even though the density reaches its maximum. Since the road has a high capacity to handle normal traffic, it is likely to be frequently used. Figure 8 shows that the speed is not 0 when the density is 1, indicating that even with a high volume, traffic is smooth with no congestion. In addition, the speed does not decrease linearly to 0 with an increase in density beyond 0.5, which differs from the Greenshields traffic model given in [32].

#### 4.3.2. Density versus Flow

**Figure 9:**
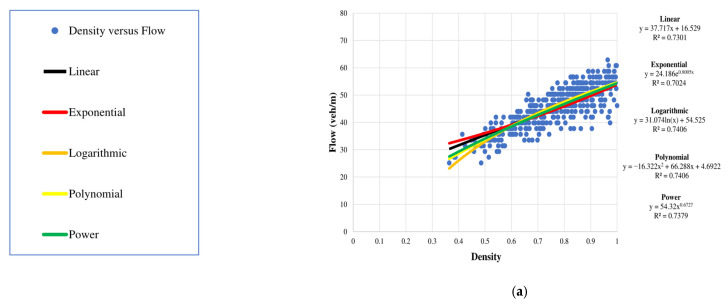

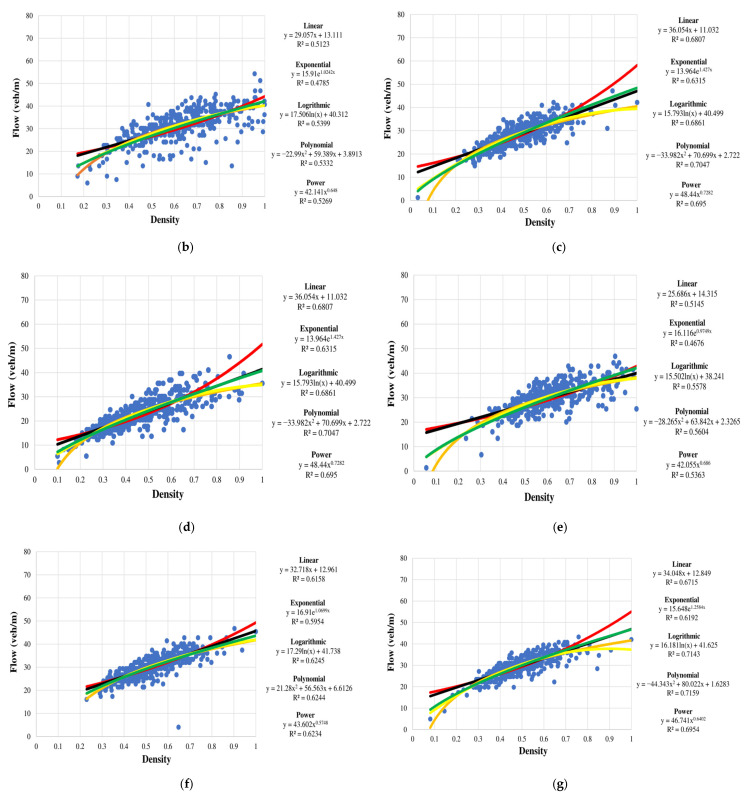
Density versus flow for the week 10 January 2022 to 16 January 2022: (**a**) Monday, 10 January 2022; (**b**) Tuesday, 11 January 2022; (**c**) Wednesday, 12 January 2022; (**d**) Thursday, 13 January 2022; (**e**) Friday, 14 January 2022; (**f**) Saturday, 15 January 2022; and (**g**) Sunday, 16 January 2022.

Figure 9 gives the relationship between density and flow for the seven days. From (13), the flow increases with density until the road capacity is reached. Once the capacity is exceeded, the flow decreases with a further increase in density. Figure 9 shows that the maximum density and maximum flow occur, which indicates that the capacity has been exceeded. However, this does not significantly affect the traffic as there is no congestion.

#### 4.3.3. Speed versus Flow

**Figure 10:**
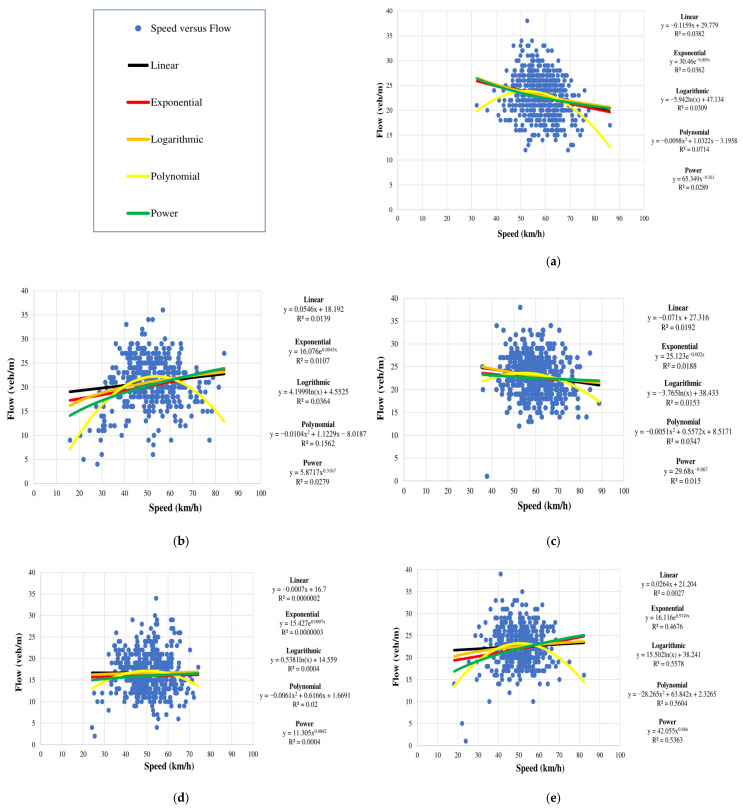

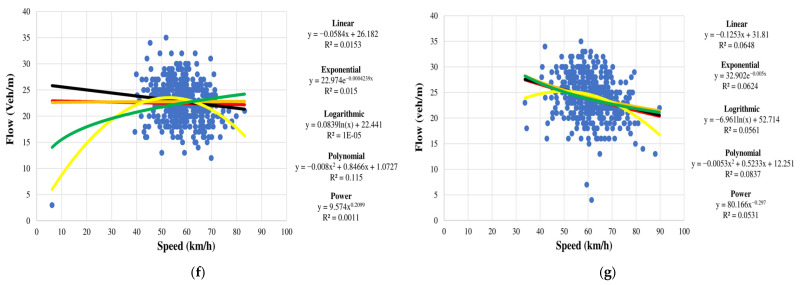
Speed versus flow for the week 10 January 2022 to 16 January 2022: (**a**) Monday, 10 January 2022; (**b**) Tuesday, 11 January 2022; (**c**) Wednesday, 12 January 2022; (**d**) Thursday, 13 January 2022; (**e**) Friday, 14 January 2022; (**f**) Saturday, 15 January 2022; and (**g**) Sunday, 16 January 2022.

The Greenshields model (6) provides the relationship between speed and flow. According to this model, when there are no vehicles on the road, i.e., zero density, the flow is also zero. As density increases, the flow also increases until it reaches its maximum at the road capacity. Beyond this point, as the density continues to increase, the flow decreases and eventually drops to zero when the density reaches its maximum. The corresponding speed is also zero. Figure 10 gives the relationship between flow and speed for the seven days. This shows that the flow is near maximum and the speed is never low. Thus, the road has a high capacity and is free of congestion. If congestion were to occur, the jam density would be reached, causing the flow and speed to drop to 0.

## 5. Conclusions

Intelligent transportation systems employ a diversity of methodologies for monitoring and aggregating traffic data. They often leverage a variety of devices including pressure sensors, piezoelectric sensors, radar systems, and pneumatic tubes. Developing robust sensor nodes is challenging due to cost, implementation complexity, limited parameter availability, and issues related to data transmission and management. These are overcome here using advanced technologies such as image processing, machine learning, and cloud storage. The proposed edge computing solution is low-cost and energy efficient. It includes a Raspberry Pi 4, Pi camera, Neural Compute Stick 2, Xiaomi MI Power Bank, and Zong 4G Bolt+. The sensor node is compact so it can easily be installed on a roadside. A key component is the MobileNet-SSD model which is used for accurate vehicle detection. The centroid tracking algorithm is used to estimate velocity. The computational complexity is low, resulting in excellent energy efficiency. Thus, the proposed sensor node is superior to existing solutions.

The system was field evaluated over 7 days for 7 h a day on a diverse range of traffic. It was able to extract vehicle count, speed, direction, and type, flow, peak hour factor, density, time headway, and distance headway for approximately 10,000 vehicles per day. During this period, there was no congestion, and the flow was smooth with high speeds. The count and speed accuracy were 93.2% and 82.9%, respectively. The power consumption of the sensor node was only 1.2 A per hour.

The Greenshields model was used to characterize the relationships between density, speed, and flow. It was shown that the flow increases with density until the road capacity is reached. The speed versus flow relationship also followed this model. The results suggest that the observed road segment has been well-designed and is capable of handling high traffic volumes without creating congestion. The parameters obtained can be used to develop models for use in traffic simulation software. The proposed edge computing solution can provide valuable insights into road traffic behavior to facilitate intelligent transportation systems and smart urban mobility development.

There are several avenues for future research. Node operation can be extended by incorporating energy harvesting, solar panels, and advanced batteries. While the pre-trained model employed has excellent performance, other models can be considered to improve accuracy, particularly in heterogeneous traffic environments. Moreover, a network of sensor nodes can be deployed to provide insights into traffic dynamics across multiple road segments and at intersections.

## Figures and Tables

**Table 1:** Traffic flow on University Road in Peshawar, Pakistan during the week 10–16 January 2022.

Day	Cars	Buses	Motorbikes	Bicycles	Animal-Drawn Carts	Total
Monday	9821	290	2041	62	23	12,237
Tuesday	7004	220	1078	75	16	8393
Wednesday	8635	244	1989	60	13	10,941
Thursday	7834	73	940	45	12	8904
Friday	7206	283	1604	145	17	9255
Saturday	7608	280	1645	51	18	9602
Sunday	8008	375	1495	61	14	9953
Total	56,116	1765	10,792	499	113	69,285
Percentage	81.0%	2.5%	15.6%	0.72%	0.16%	100%

**Table 2:** Proposed system performance.

	Manual	System	Difference	Error	Accuracy
Vehicle Count	2356	2196	160	6.8%	93.2%
Average Speed (km/h)	54.9	64.3	±9.4	17.1%	82.9%

**Table 3:** Speed (km/h), flow (veh/m), and density (veh/km) statistics for the seven days.

Day	Parameter	Min	First Quartile	Second Quartile	Third Quartile	Max	Mean	Standard Deviation
1	Speed	32.1	53.9	58.6	63.4	86.1	58.4	7.0
	Flow	12.0	20.0	23.0	24.0	38.0	23.0	4.0
	Density	10.4	19.9	23.7	27.7	43.3	24.0	5.7
2	Speed	15.8	44.6	51.3	58.0	83.8	51.3	10.6
	Flow	4.0	18.0	21.0	24.0	36.0	21.0	4.9
	Density	6.8	20.5	24.9	29.8	48.6	25.4	7.3
3	Speed	35.7	54.9	60.4	66.0	88.7	60.0	8.3
	Flow	12.0	20.0	23.0	26.0	32.0	23.1	4.1
	Density	10.1	19.0	22.5	26.5	48.3	23.6	5.8
4	Speed	24.1	44.6	50.8	56.6	74.1	50.9	8.7
	Flow	4.3	20.0	22.0	25.0	34.0	16.6	4.6
	Density	4.3	22.7	26.7	31.5	43.8	20.2	6.7
5	Speed	18.1	53.1	57.8	63.2	82.1	50.3	8.8
	Flow	5.0	20.0	23.0	25.0	39.0	22.5	4.3
	Density	10.5	20.5	23.7	27.8	56.9	27.6	7.4
6	Speed	6.1	53.1	57.8	63.2	83.1	58.2	8.1
	Flow	3.0	20.0	23.0	25.0	35.0	22.7	3.8
	Density	10.3	20.5	23.7	27.8	44.9	23.9	5.5
7	Speed	33.6	55.9	61.0	67.1	89.7	61.3	8.4
	Flow	4.0	22.0	24.0	27.0	35.0	24.1	4.2
	Density	3.9	20.0	23.9	27.9	48.5	24.1	6.0

## Data Availability

Not applicable.
